# Pyrin variant E148Q potentiates inflammasome activation and the effect of pathogenic mutations in *cis*

**DOI:** 10.1093/rheumatology/kead376

**Published:** 2023-07-22

**Authors:** Thomas Reygaerts, Pawat Laohamonthonkul, Katja Hrovat-Schaale, Fiona Moghaddas, Paul J Baker, Paul E Gray, Seth L Masters

**Affiliations:** Inflammation Division, The Walter and Eliza Hall Institute of Medical Research, Parkville, VIC, Australia; Department of Medical Biology, The University of Melbourne, Parkville, VIC, Australia; Inflammation Division, The Walter and Eliza Hall Institute of Medical Research, Parkville, VIC, Australia; Department of Medical Biology, The University of Melbourne, Parkville, VIC, Australia; Inflammation Division, The Walter and Eliza Hall Institute of Medical Research, Parkville, VIC, Australia; Department of Medical Biology, The University of Melbourne, Parkville, VIC, Australia; Immunology and Allergy Centre, North Bristol NHS Trust, Bristol, UK; Inflammation Division, The Walter and Eliza Hall Institute of Medical Research, Parkville, VIC, Australia; Department of Medical Biology, The University of Melbourne, Parkville, VIC, Australia; Department of Medicine, University of Western Sydney, Campbelltown, NSW, Australia; Department of Immunology and Infectious Diseases, Sydney Children’s Hospital, Randwick, NSW, Australia; Inflammation Division, The Walter and Eliza Hall Institute of Medical Research, Parkville, VIC, Australia; Department of Medical Biology, The University of Melbourne, Parkville, VIC, Australia

**Keywords:** pyrin, inflammasome, FMF, IL-1, cytokines, polymorphism, risk factor

## Abstract

**Objective:**

The p.E148Q variant in pyrin is present in different populations at a frequency of up to 29%, and has been associated with diseases, including vasculitis and FMF. The pathogenicity of p.E148Q in FMF is unclear, even when observed *in cis* or *in trans* to a single, typically recessive, pathogenic mutation. We performed functional validation to determine whether p.E148Q increases the ability of pyrin to form an active inflammasome complex in cell lines.

**Methods:**

We interrogated the Australian Autoinflammatory Disease RegistrY (AADRY) to find candidate inheritance patterns for the p.E148Q variant in pyrin. Different pyrin variant combinations were tested in HEK293T cells stably expressing the adaptor protein apoptosis-associated speck-like (ASC), which were analysed by flow cytometry to visualize inflammasome formation, with and without stimulation by *Clostridioides difficile* toxin B (TcdB). Inflammasome-dependent cytokine secretion was also quantified by ELISA of supernatants from THP-1 cells transduced with lentiviral expression vectors.

**Results:**

In AADRY, we observed the p.E148Q allele in individuals with autoinflammatory diseases alone or in conjunction with other pyrin variants. Two FMF families harboured the allele p.E148Q-M694I *in cis* with dominant heritability. *In vitro*, p.E148Q pyrin could spontaneously potentiate inflammasome formation, with increased IL-1β and IL-18 secretion. p.E148Q *in cis* to classical FMF mutations provided significant potentiation of inflammasome formation.

**Conclusion:**

The p.E148Q variant in pyrin potentiates inflammasome activation *in vitro*. *In cis*, this effect is additive to known pathogenic FMF mutations. In some families, this increased effect could explain why FMF segregates as an apparently dominant disease.

Rheumatology key messages

- A common variant in pyrin (E148Q) can increase inflammasome activation.
- Increased inflammasome activation of pyrin E148Q is additive to known pathogenic mutations that cause FMF.
- Pyrin E148Q *in cis* to known pathogenic mutations may explain some dominant inheritance of FMF.

## Introduction

The protein pyrin is mutated in the disease FMF. This is the prototype autoinflammatory disease (AID), which presents as recurrent fever, serositis and arthritis [1]. The classical FMF mutations found in the B30.2 domain are gain-of-function mutations [2], and FMF is genetically confirmed when the patient presents with one pathogenic mutation on each allele [3]. However, the classical clinical picture is sometimes complicated by a variable expressivity and penetrance, and many cases are not fully supported by a genetic diagnosis. Indeed, only about 20–40% of the FMF cases can be genetically confirmed, and heterozygous cases are seen about 30% of the time, despite extensive searches for a second mutation [4]. It is also recognized that individuals with one heterozygous allele can show atypical FMF or an inflammatory signature in the blood when not having a classical FMF disease [5]. This situation is highly compatible with the idea of FMF being of variable expressivity for individuals with a heterozygous pathogenic mutation. For many of these cases, a common variant in pyrin can be observed, p.Glu148Gln (p.E148Q). However, the pathogenicity of this allele is highly debated in the literature.

The p.E148Q allele is found at a high prevalence in healthy individuals from several populations (29%, 5.5% and 1.3–1.4% in East/South Asian, Askhenazi Jewish, and African/European populations, respectively, gnomad.broadinstitute.org). The physiological effect of this variant is thus predicted to be small, which makes difficult to perform functional studies. Still, numerous FMF cases with the p.E148Q polymorphism *in cis* or *in trans* of other pathogenic mutations are described in the literature, and the question of its significance is relevant for many patients. Some papers described FMF patients with p.E148Q in the homozygous state or associated with another FMF mutation presenting less severe pathology than recessively inherited B30.2 domain mutations [6]. Complex alleles with p.E148Q found *in cis* of other mutations are registered in the Infevers database (infevers.umai-montpellier.fr, can be found with p.L110P, p.S179N, p.P369S, p.P369S and p.R408Q, p.I692del, p.M694V, p.M694I, p.V7126A, p.A744S, p.R761H, p.V726A and p.R761H) and seem to be sufficient in the heterozygous state to cause FMF in some individuals [7]. Observational studies have shown that p.E148Q could potentially increase the effect of moderate alleles such as p.V726A and p.R761H [8, 9]*.* A recent penetrance study in 148 patients referred for FMF found a penetrance 17 times higher for the genotype [(E148Q)];[(M694V)] (p.E148Q and p.M694V *in trans*) than [=];[(M694V)] (p.M694V heterozygous) (0.135 and 0.008, respectively) [10]. Also two recent, independent studies support that p.E148Q could influence the severity of FMF in individuals with a heterozygous B30.2 domain mutation and increase the level of IL-18 cytokine expression at remission in those individuals [11, 12]. Finally, a recent report evaluated the genotype of 169 FMF patients with a concurrent diagnosis of AA amyloidosis. Unexpectedly, 14.8% of those patients had the genotype [(E148Q)];[(M694I)] and 11.4% [(E148Q)];[(E148Q)] (p.E148Q homozygous) [13]. From this evidence in the literature, we suspect that p.E148Q could act as a disease modifier, particularly in combination with other classical FMF mutations.

Patient cohorts have also shown that FMF mutation carriers could be predisposed to other inflammatory diseases independently of FMF [14]. p.E148Q specifically has been associated with IgA vasculitis [15, 16], Ulcerative Colitis and the need for surgery [17], Crohn’s disease and perianal injury [18], a stricturing disease pattern, and extra-intestinal disease [19]. It has also been shown to increase the risk of developing AA amyloidosis alone or in combination with other autoinflammatory diseases (AID) causing mutations [20]. Moreover, it has been linked to Behçet’s disease [21] and recurrent aphthous stomatitis [22], RA severity [23], and multiple sclerosis [24].

At the molecular level, pyrin oligomerizes with the apoptosis-associated speck-like (or ASC) protein to form a complex called the inflammasome. This allows the activation of caspase-1 and the secretion of IL-1β and IL-18 [25]. Pyrin inflammasome activation has been demonstrated for several known pathogenic mutations; however, a lack of functional evidence for the variant p.E148Q prompted us to evaluate its ability to activate the pyrin inflammasome alone and in combination with classical B30.2 domain FMF mutations.

## Materials and methods

### Patients

The Australian Autoinflammatory Diseases RegistrY AADRY database (aadry.org) was interrogated for carriers of the p.E148Q variant in pyrin and their clinical data. All the patients have consented to the use of their clinical information. Ethical approval was granted by committees of the Monash Health: HREC/15/MonH/31 and the Sydney Children’s Hospitals Network: HREC/15/SCHN/346.

#### Patient and public involvement

Patients were recruited to AADRY based on clinical presentation consistent with an autoinflammatory disease. Several patients harbour the p.E148Q allele, and indicated that they would like more information regarding the potential pathogenicity of this variant. Although patients/public were not involved in the design, conduct or outcome measures of the study, they will be involved in dissemination of the published study results via linked communities such as the FMF & AID Global Association australia@fmfandaid.org and at public lectures.

### Cell culture

HEK293T cells were cultured in DMEM supplemented with 10% foetal calf serum (FCS), 1% penicillin and streptomycin. HEK293T cells stably expressing ASC tagged with enhanced green fluorescent protein (EGFP) were generated by transfection using Lipofectamine2000 (Invitrogen, 11668–019) according to manufacturer’s instructions. THP-1 cells were cultured in Roswell Park Memorial Institute medium (RPMI) supplemented with 10% FCS, 1% penicillin and streptomycin. *MEFV* knock-out THP-1 cells were generated by Clustered Regularly Interspaced Short Palindromic Repeats (CRISPR)/Cas9 technology, as previously described [26].

### Plasmids and site-directed mutagenesis

Expression and lentiviral vectors encoding pyrin tagged with mCherry or glutathione S-transferase (GST) have been described elsewhere [27]. Wild-type (WT), p.S242R and p.M694V pyrin constructs were created for previous studies [27]. Site-directed mutagenesis was performed according to the manufacturer’s instruction with the QuikChange Lightning Multi Site-Directed Mutagenesis Kit (catalog #210514, Agilent Technologies, Santa Clara, CA, USA). Mutations were introduced using the following primers:

L110P (c.t329c)

5′-gcgtccagctccccgggggagaacaag-3′

5′-cttgttctcccccggggagctggacgc-3′

E148Q (c.g442c)

5′-cctcccggcctggggctggctgc-3′

5′-gcagccagccccaggccgggagg-3′

R202Q (c.g605a)

5′-gtttctgcgcagctggacctcggcctg-3′

5′-caggccgaggtccagctgcgcagaaac-3′

M694I (c.g2082a)

5′-ggctactgggtggtgataatgataaaggaaaatgagtacc-3′

5′-ggtactcattttcctttatcattatcaccacccagtagcc-3′

V726A (c.t2177c)

5′-aaaaggagatgcttccagctctgtagtccacgaag-3′

5′-cttcgtggactacagagctggaagcatctcctttt-3′

R761H (c.g2282a)

5′-tcttccctccatcatgtgtcccagggctg-3′

5′-cagccctgggacacatgatggagggaaga-3′

### THP-1 cells lentiviral infection and stimulation

Lentivirus was produced as previously described [28]. One million THP-1 cells *MEFV* KO were infected using 3 ml of virus and 7 μg of polybrene. Plates were centrifuged at 800 g for 3 h at 32°C and then incubated at 37°C. Cells were washed in Dulbecco’s PBS (DPBS, Gibco) 24 h after infection and seeded 48 h after infection with or without Pam3CSK4 (500 ng/ml, InvivoGen, San Diego, CA, USA, 112208–00-1) and native *Clostridioides difficile* Toxin B protein at various concentrations (TcdB, ab124001, Cambridge, UK). Cells were lysed and immunoblotted for protein expression. IL-1β and IL-18 were measured in culture supernatants by ELISA 24 h after seeding (R&D Systems, DY201 and DY318-05).

### Flow cytometry

ASC Speck assay (or time of flight inflammasome assay) was performed as previously described [29]. Briefly, 0.75 × 10^5^ HEK293T cells stably expressing ASC tagged with EGFP were seeded in a 24-well plate before transfection of 50 ng of expression vectors encoding WT or mutant pyrin tagged with mCherry using Lipofectamine 2000 (Invitrogen, 11668–019) according to the manufacturer’s instructions in OptiMEM I (reduced Serum Medium, Gibco). Quantification of ASC specks in the cells using flow cytometry was performed 16 h after transfection, or 20 h if the cells were stimulated by TcdB (10 ng/ml 4 h before harvesting). The gating strategy is presented in Supplementary Fig. S1 (available at *Rheumatology* online). Briefly, cells expressing the same level of pyrin tagged with mCherry and showing 10 or 20% of ASC specks in cells expressing WT pyrin were analysed in all the conditions. This allowed control for inter-experimental variability.

### Immunoprecipitation and western blotting

1.5 × 10^6^ HEK293T cells were seeded in 10-cm^2^ dishes before being transfected with 5 μg of WT or mutant pyrin tagged with GST. Cells were lysed on ice 48 h after transfection using 1% NP-40 lysis buffer supplemented with protease inhibitors [1% NP-40 (Sigma-Aldrich, CA-630), 2 mM EGTA, 150 mM NaCl, 20 mM Tris-HCl pH 7.4, 4% glycerol, 5 mM NaF, 10 mM NaPPi, 1 mM PMSF, 1 mM sodium vanadate (Na_3_VO_4_), 1×  cOmplete protease inhibitors (Roche, #11697498001), Nuclease-free water). Immunoprecipitations of pyrin were performed with glutathione-coated sepharose beads 4B (GE Healthcare). THP-1 cells were lysed using 1% NP-40 lysis buffer 48 h after lenti-viral transduction. Immunoblotting was performed using 4–12% NuPAGE Bis-Tris gels (Invitrogen, Carlsbad, CA, USA) in MES running buffer. Protein transfers were done on nitrocellulose membrane. Membranes were blocked using 5% of BSA in tween/tris-buffered saline buffer (TBST) and probed overnight with the primary antibody at 4°C before using the secondary antibody. All antibodies were eluded with 1% BSA in TBST. Experiments were performed using the following antibodies; pyrin (1:500 AdipoGen #AL196), actin (1:10.000 anti-β-actin HRP, Santa Cruz, sc-47778) and pan-14–3-3 (1:500, Santa Cruz, sc-1657).

### Statistical analysis

Two-tailed *t*-tests were performed for the analysis of ASC speck data. The Mann–Whitney non-parametric test was used for the ELISA analysis. Such tests were performed using Prism software (GrapPad Software, La Jolla, CA, USA). Data are represented as mean (s.e.m.) for the ASC-speck data and as mean (s.d.) for the ELISA data. (*P <0.05, **P <0.01, ***P <0.001, unless otherwise specified).

## Results

### p.E148Q pyrin in the Australian Autoinflammatory Disease RegistrY

**Figure 1.**
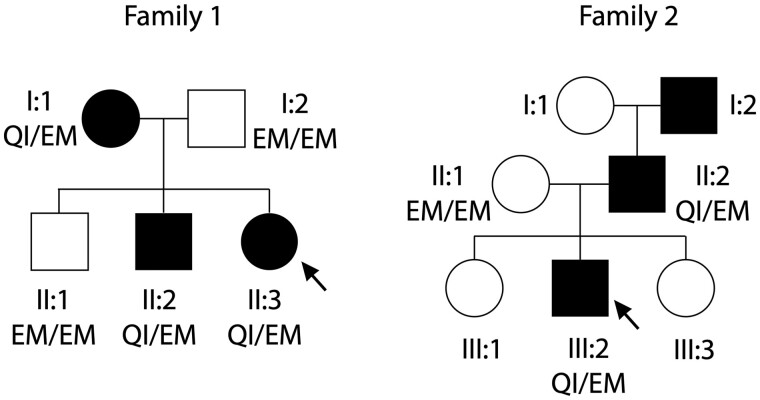
p**.**E148Q *in cis* with p.M694I segregates with disease in a dominant fashion for two families in Australian Autoinflammatory Diseases RegistrY (AADRY). Family pedigrees found in the AADRY presenting the allele p.E148Q-M694I *in cis* in the heterozygous state. The genetic background is Lebanese (family 1) and Egyptian (family 2). The closed boxes represent affected individuals; the open boxes represent unaffected individuals. Patients who have been sequenced have their genotype represented in the graph. QI/EM represents the genotype [(E148Q; M694I)];[=], corresponding to the amino acid changes of glutamic acid to glutamine at position 148 and methionine to isoleucine at position 694 *in cis* of a wild-type (WT) allele. EM/EM represents the genotype [=];[=], corresponding to two WT alleles. Although not showing a confirmed FMF genetic result (pathogenic mutation in the B30.2 domain on the two alleles), all the individuals presenting the complex allele p.E148Q-M694I in the heterozygous state had a clinically typical FMF (fulfilling the Tel Hashomer criteria). The family trees suggest a dominant inheritance of FMF

AADRY is a registry of patients with AID. For those without a known genetic cause, whole-exome or genome sequencing is performed preferably as a trio (a patient with his parents), with the aim of identifying pathogenic or novel variants of interest. From 140 AID patients and 225 healthy relatives, 10 AID patients and one healthy individual harboured the variant p.E148Q. Four patients presented recurrent symptoms in the AID spectrum, although they did not have a clinical designation as FMF. The p.E148Q allele was present heterozygously in one of those patients and combined with other variants in the *MEFV* gene for the other three (two of them presenting one pathogenic mutation *in cis* of p.E148Q). One healthy relative presented p.E148Q homozygously. Six patients presented with classical FMF fulfilling the Tel Hashomer criteria and segregated in two families (one of Lebanese and one of Egyptian origin). They presented the rare genotype [=];[(E148Q; M694I)], corresponding to the amino acid changes glutamic acid to glutamine at position 148 and methionine to isoleucine at the position 694 *in cis* of a WT allele, with a familial pattern suggesting dominance inheritance (Fig. 1). Their clinical histories are presented in Supplementary Data S1, available at *Rheumatology* online.

### Spontaneous inflammasome activation due to p.E148Q pyrin

**Figure 2.**
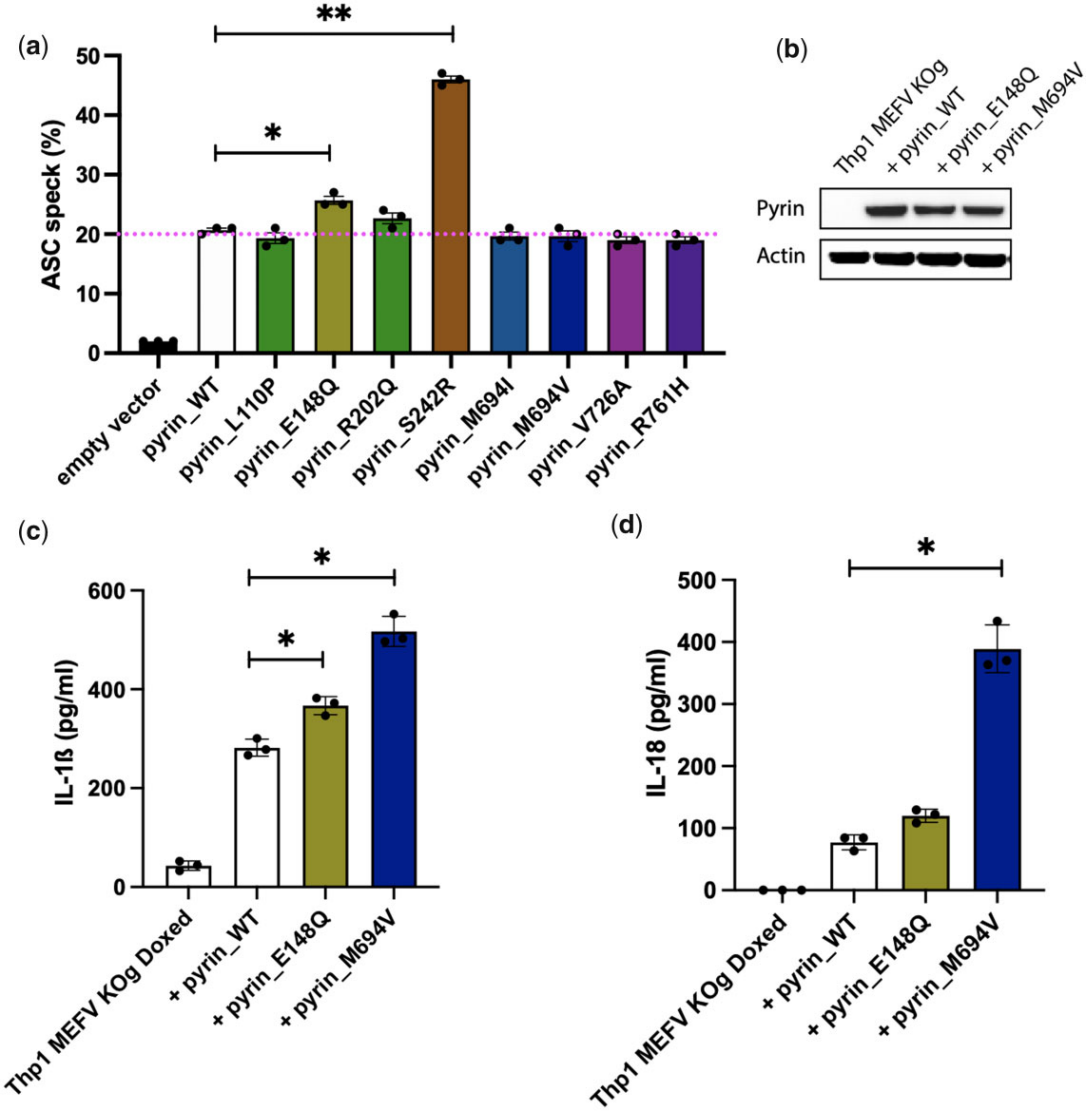
p.E148Q pyrin promotes ASC speck formation and cytokine expression. **(a)** ASC speck assay in HEK293T cells stably expressing ASC EGFP and transiently transfected with mCherry-tagged WT pyrin, p.L110P, p.E148Q, p.R202Q, p.S242R, p.M694I, p.M694V, p.V726A and p.R761H. We used a gating strategy selecting mCherry-expressing cells to get 20% of ASC specks in the WT-transfected cells. Data are pooled from three independent experiments. Error bars represent mean ± s.e.m.. Paired Student’s *t*-test *P<0.05, **P<0.001. HEK293T cells transfected with p.E148Q pyrin showed an increase in ASC speck compared with WT pyrin. (**b**) Western blot analysis of the pyrin expression in the transduced THP-1 cells (shown in **c** and **d**) as a representative result of three independent experiments. (**c**) hIL-1β cytokine secretion of Cas9 *MEFV* KO THP-1 cells after lenti-viral transduction of WT pyrin, p.E148Q and p.M694V and priming with Pam3CSK4 for 24 h. (**d**) hIL-18 cytokine secretion. Cells were handled as in (**c**) but were not primed with Pam3CSK4. In (**c**) and (**d**), error bars represent mean (s.d.). Graphs show a representative result of three independent experiments. Mann–Whitney one-tailed test. * in (**c**) and (**d**) represent a *P*-value of 0.05. p.E148Q pyrin potentiate IL-1β and IL-18 cytokine secretion in THP-1 cells

To evaluate whether p.E148Q pyrin has any effect favouring inflammasome formation, we evaluated this polymorphism in an ASC speck assay [29]. Unstimulated HEK293T cells stably expressing ASC tagged with EGFP were transfected with WT or mutated pyrin tagged with mCherry. Using flow cytometry, we could evaluate the intracellular reorganization of ASC from a homogeneous cytoplasmic distribution to the speck-like structure when an inflammasome complex is formed. Flow cytometry allowed selection of cells with a comparable level of pyrin expression (see gating strategy in Supplementary Fig. S1, available at *Rheumatology* online). Using this assay, there were more cells with a speck in p.E148Q pyrin-transfected cells than WT pyrin-transfected cells (Fig. 2a). The difference was modest compared with the effect of the p.S242R pyrin mutation, which causes Pyrin-Associated Autoinflammation with Neutrophilic Dermatosis (PAAND) by disrupting the inhibition of pyrin by 14–3-3, and is known to be constitutionally active in this assay [27]. The mutations in the B30.2 domain of pyrin p.M694V, p.M694I, p.V726A and p.R761H did not show an elevated number of cells with an inflammasome speck, nor did the variants of undetermined significance p.L110P and p.R202Q located in exon 2 of pyrin near the 148 amino acid position (Fig. 2a). We then took advantage of the monocytic cell line THP-1 and lentivirally transduced them with WT or mutated pyrin expression constructs. p.E148Q pyrin increased the level of secreted IL-1β after priming of the cells by Pam3CSK4 (Fig. 2c), and increased the secretion of IL-18 without any priming of the cells (Fig. 2d). In that system, p.M694V pyrin overexpression showed enhanced cytokine expression (Fig. 2c and d). In summary, p.E148Q pyrin constructs seem to potentiate inflammasome formation and cytokine expression spontaneously compared with pyrin WT.

### Stimulation of pyrin potentiates B30.2 domain mutations, but not p.E148Q

**Figure 3.**
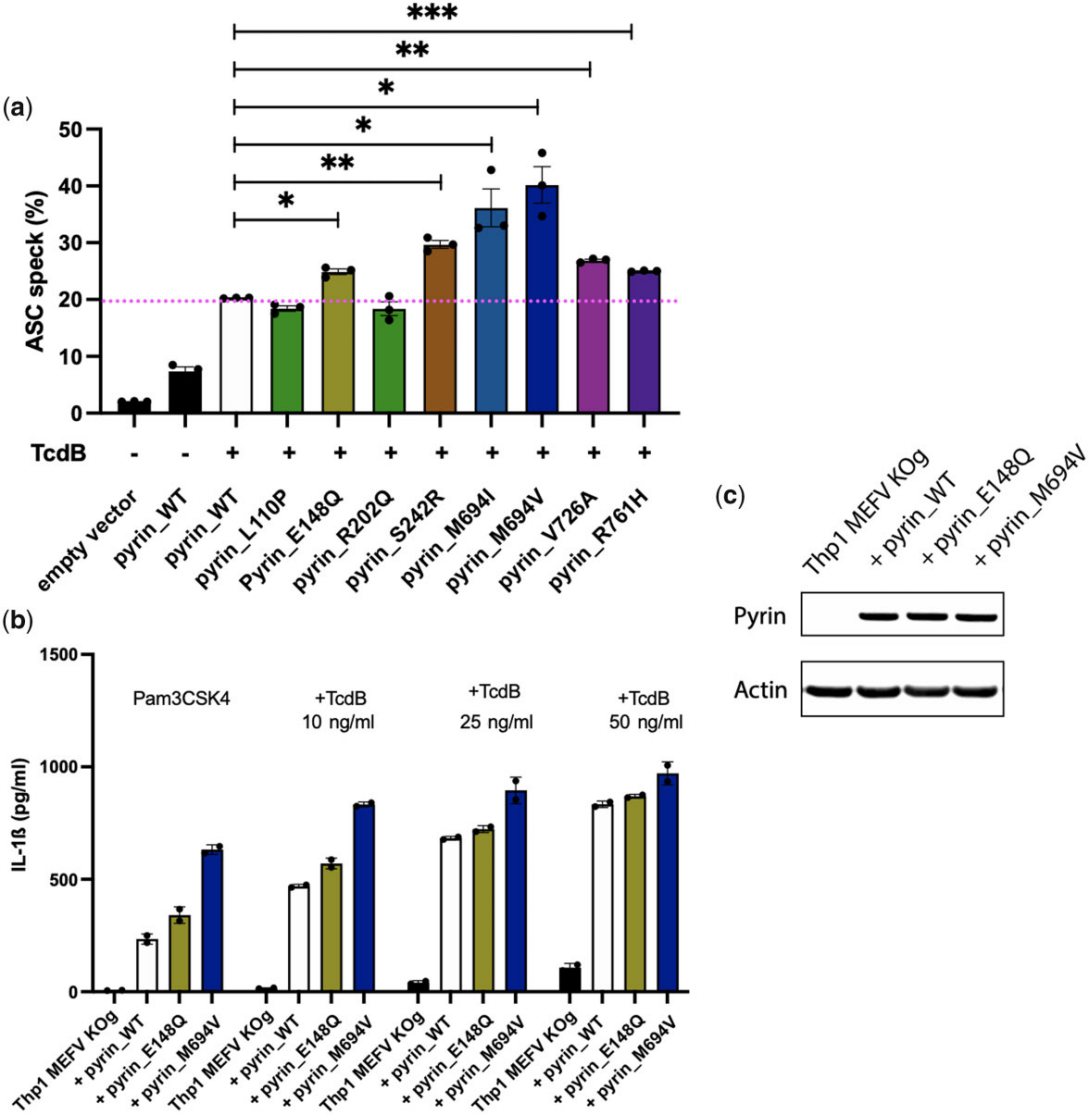
Stimulation of p.E148Q pyrin by TcdB does not promote more ASC speck formation and cytokine expression. (**a**) ASC speck assay in HEK293T cells stably expressing ASC EGFP and transiently transfected with mCherry-tagged WT pyrin, p.E148Q, p.M694I, p.M694V, p.V726A and p.R761H, with and without TcdB stimulation. We used a gating strategy selecting mCherry-expressing cells to get 20% of ASC specks in the WT-transfected cells stimulated. Data are pooled from three independent experiments. Error bars represent mean (s.e.m.). Paired Student’s *t*-test *P<0.05, **P<0.01, ***P<0.001. TcdB stimulation of p.E148Q pyrin did not promote further ASC speck inflammasome formation, the amount seen in this condition being comparable with that in the unstimulated condition. This situation contrasted with the FMF mutations found in the B30.2 domain. (**b**) hIL-1β cytokine secretion of Cas9 *MEFV* KO THP-1 cells after lenti-viral transduction of WT pyrin, p.E148Q and p.M694V constructs, without and with increasing TcdB stimulation. Error bars represent mean (s.d.). Graphs show a representative result of three independent experiments. Mann–Whitney one-tailed test. TcdB stimulation did not increase cytokine expression further than the increase seen without stimulation. (**c**) Western blot analysis of the pyrin expression of the THP-1 cells shown in (**b**) as a representative result of three independent experiments

Pyrin can be stimulated by various triggers, which may provide important context for disease penetrance. As a model trigger, we stimulated pyrin *in vitro* with TcdB, which inactivates the small GTPase RhoA, stopping PKN1/2 phosphorylation of pyrin and its inhibition by 14–3-3 [30]. Using the same HEK293T cell assay of inflammasome formation as in Fig. 2, we further stimulated the cells with TcdB. We found again an increase for pyrin p.E148Q compared with pyrin WT (Fig. 3a). The level of increase of the percentage of cells presenting an ASC speck appears similar to the experiment with the unstimulated cells (Fig. 2a). Under those conditions, the B30.2 domain mutations now generated a significantly increased level of inflammasome formation (Fig. 3a). Interestingly, some B30.2 mutations were more potent than others and reflected the literature, p.M694V and p.M694I seeming more potent than p.V726A and p.R761H. Stimulation of THP-1 cells with TcdB did increase the level of IL-1β produced by the transfected cells. THP-1–expressing p.E148Q pyrin secreted more IL-1β compared with WT pyrin when stimulated by Pam3CSK4 and at low TcdB concentration. Interestingly, the amount of IL-1β produced by p.E148Q pyrin–transfected cells did not increase with TcdB, showing that p.E148Q works independently of TcdB stimulation. At higher TcdB concentrations, IL-1β rapidly plateaued, as shown by the IL-1β secretion from p.M694V pyrin–expressing cells (Fig. 3b). Data from Figs 2 and 3 suggest that the mechanism for inflammasome potentiation by p.E148Q is spontaneous and independent of pyrin dephosphorylation by TcdB.

Currently, the best described mechanism by which pyrin mutations operate is prevention of inhibitory 14–3-3 binding, as in the case of the p.S242R mutation causing PAAND [31]. To examine whether the p.E148Q variant functions in the same way, we immunoprecipitated overexpressed pyrin with a GST tag in HEK293T cells and evaluated 14–3-3 binding via western Blot (Supplementary Fig. S2, available at *Rheumatology* online). Contrary to p.S242R mutation, p.E148Q did not alter 14–3-3 binding to pyrin. These data would suggest that the polymorphism p.E148Q increases pyrin inflammasome formation via a mechanism that is different to the B30.2 domain mutations, and independent of 14–3-3 binding.

### p.E148Q potentiates pyrin activation due to other FMF mutations

**Figure 4.**
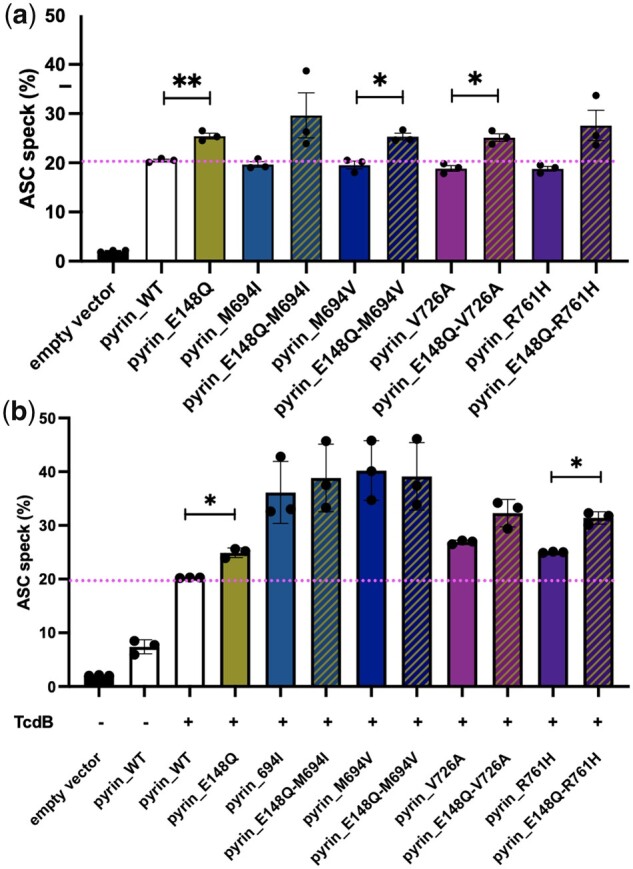
p.E148Q promotes further ASC speck formation of FMF-mutated pyrin. (**a**) ASC speck assay in HEK293T cells stably expressing ASC EGFP and transiently transfected with mCherry-tagged WT pyrin, p.E148Q, p.M694I, p.M694V, p.V726A and p.R761H and the combined mutation p.E148Q-M694I, p.E148Q-M694V, p.E148Q-V726A and p.E148Q-R761H. We used a gating strategy selecting mCherry-expressing cells to get 20% of ASC specks in the WT-transfected cells. If combined *in cis* with classical FMF mutation, p.E148Q promoted ASC speck inflammasome formation spontaneously. (**b**) Cells transfected as in (**a**) were stimulated with TcdB. We used a gating strategy selecting mCherry-expressing cells to get 20% of ASC specks in the WT-transfected cells stimulated. Data are pooled from three independent experiments. Error bars represent mean (s.e.m.). Paired Student’s *t*-test *P<0.05, **P<0.01. With stimulation, alleles presenting p.E148Q *in cis* of moderate pathogenic mutations showed more ASC specks, and a trend if combined with more potent mutations

Individuals with clinical FMF in AADRY showed p.E148Q in combination with p.M694I on the same allele (*in cis*). As p.E148Q can potentiate pyrin inflammasome formation, we were interested to study p.E148Q in combination with other mutations to see whether p.E148Q could act as a modifier of the effect of classical FMF mutations. Studying inflammasome formation in HEK293T cells without TcdB stimulation showed an increase in ASC speck formation when p.E148Q was added to classical FMF pathogenic mutations. This increased effect was similar for various B30.2 domain mutations combined with p.E148Q, reaching significance for p.M694V and p.V726A (Fig. 4a). Stimulating those cells with TcdB showed a trend towards inflammasome formation when p.E148Q was combined with pathogenic FMF mutations for p.M694I and p.V726A. The combination with p.R761H reached statistical significance (Fig. 4b). The increased percentage of cells presenting an ASC speck in p.E148Q pyrin cells compared with WT pyrin was comparable with the level of increase if p.E148Q was added to the B30.2 domain mutations p.V726A and p.R761H. For the more potent p.M694I and p.M694V, adding p.E148Q was of little effect (Fig. 4b). In summary, p.E148Q seems to promote ASC speck formation with and without TcdB stimulation, so its gain of function is constitutive. In stimulated cells, p.E148Q increases speck formation even further in addition to some B30.2 domain mutations, although this may become saturated for the strongest B30.2 domain mutations in our assay.

### Heterozygous p.E148Q potentiates inflammasome formation *in cis* with p.M694I

**Figure 5.**
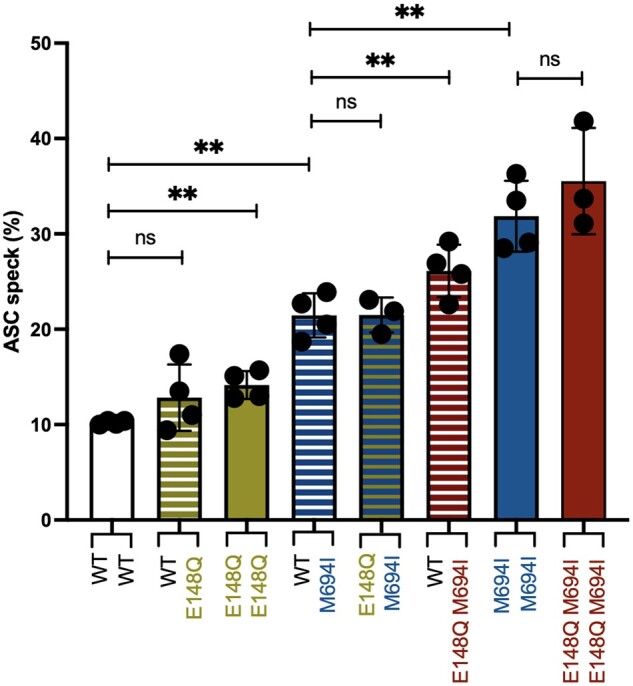
Heterozygous p.E148Q *in cis* with p.M694I potentiates the effect of p.M694I in response to TcdB. HEK293T cells stably expressing ASC EGFP and transiently co-transfected with mCherry tagged WT pyrin, p.M694I and p.E148Q-M694I and stimulated with TcdB. FACS analysis revealed a statistically increased ASC speck formation for cells co-expressing p.E148Q *in cis* of p.M694I and the WT allele (E148Q *cis* M694I), compared with the cells expressing p.M694I pyrin and the WT allele (M694I heterozygous). We used a gating strategy selecting mCherry-expressing cells to get 10% of ASC specks in the WT-transfected cells stimulated. Data are pooled from four independent experiments. Error bars represent mean (s.e.m.). Paired Student’s *t*-test **P<0.01, ns; non-significant. Heterozygous p.E148Q potentiated the effect of p.M694I if it was present *in cis*

Finally, as our patients in AADRY harboured the genotype [=];[(E148Q; M694I)], we wanted to interrogate the effect of the WT allele and the effect of p.E148Q *in cis* or *in trans* position of the pathogenic mutation p.M694I. We again took advantage of our ASC speck HEK293T cell model and assayed inflammasome formation in cells co-expressing various alleles along with TcdB stimulation, as it is in those conditions that the B30.2 domain mutations showed their effect. In this experiment, p.E148Q potentiated inflammasome formation, mainly if positioned *in cis* of p.M694I (Fig. 5). Interestingly, this was the genotype presented by the families identified in AADRY (Fig. 1). p.E148Q *in trans* did not increase pyrin inflammasome formation significantly. Overall, WT pyrin did not seem to have a role in the oligomerization of the pyrin inflammasome in conjunction with pyrin-harbouring p.E148Q or p.E148Q-M694I. p.E148Q can possibly potentiate the effect of the pathogenic mutation p.M694I if found *in cis* of this mutation, a situation which parallels the case of the families identified in AADRY and cases found in the literature.

Overall, our data suggest that the p.E148Q variant in pyrin has a constitutional effect on pyrin inflammasome formation, additive to the effect of the B30.2 domain mutations in both unstimulated and stimulated conditions.

## Discussion

There is a large body of literature suggesting that p.E148Q in pyrin is a polymorphism with a functional effect, impacting the presentation of FMF or being a risk factor for other inflammatory diseases. Indeed, the allele is found in two families in AADRY, *in cis* with p.M694I and segregating with the disease as a dominant inheritance pattern. However, some arguments exist against a functional effect of this variant. For example, p.E148Q can be found at high frequencies in some populations, its frequency in some groups of healthy individuals even outmatching the frequency in FMF patients. Additionally, analysis of FMF families presenting the p.E148Q allele combined with other mutations found that it did not always segregate with disease [32]. Therefore, we must be cautious in how we interpret our data demonstrating a functional effect of p.E148Q *in vitro*.

Given that the functional effect of p.E148Q on pyrin activation we observed *in vitro* is weak compared with pathogenic mutations, it remains possible that it does not have a prominent physiologic role. However, this finding is consistent with the association of p.E148Q as a risk factor for disease, rather than acting as a monogenic mutation. Interestingly, evidence does exist concerning the efficacy of colchicine and anti-IL-1 drug therapy in some of the diseases associated with p.E148Q. For example, anti-IL-1 therapy and colchicine are used in Behçet’s disease for patients with mucocutaneous presentations, arthritis, and uveitis [33]. Also, colchicine is efficacious in relapsing/recurrent leucocytoclastic vasculitis and IgA vasculitis [34]. Furthermore, there are conditions in which IL-1β or pyrin are implicated, where p.E148Q could add to a polygenic risk score and identify a subset of patients who may respond to targeted therapy. These include IBD [36–39], systemic JIA [35] and SpAs [40].

As p.E148Q did not seem to influence pyrin’s interaction with 14–3-3, we propose that there should be an alternate mechanism to explain its functional effect. Some recent literature points towards actin polymerization being one such potential mechanism. This is because there are mutations in WDR1 [41], PSTPIP1 [42] and CDC42 [43] that are all linked to the actin cytoskeleton, and all drive pyrin-dependent autoinflammatory disease. A recent literature report suggests that CDC42 could regulate the pyrin inflammasome with a yet unknown mechanism [44]. While it is conceivable that p.E148Q may promote nucleation of the pyrin inflammasome via actin-related machinery, we have no evidence for this currently.

p.E148Q can potentiate pyrin inflammasome formation if associated with other FMF pathogenic mutations. This is relevant for patients harbouring p.E148Q *in cis* or *in trans* of other pathogenic FMF mutations. However, given that most individuals with p.E148Q homozygous, heterozygous, or in conjunction with pathogenic FMF mutations will not develop FMF [45], our data should be interpreted as indicating an increased risk of developing disease or increased severity of the disease. In some families, this increased risk can probably explain why FMF could segregate as an apparently dominant disease. Our data demonstrates that variants not considered as meaningful and nowadays discarded as benign could potentially modify the effect of other variants. This is again an evidence to not delay diagnosis and treatment of genetically heterozygous FMF patients.

As p.E148Q is present at a high frequency in some populations, yet potentially has a functional effect triggering inflammation, it is tempting to ask whether it could confer some selective advantage. P.M694V and p.V726A have both been positively selected as offering a selective advantage against *Yersinia pestis* during the successive plagues [46], but there was no evidence for an effect on p.E148Q. Indirectly, a cohort study showed that carriers of p.E148Q had increased longevity [47]; however, a specific selective advantage was not identified. It is interesting to note in our data that p.E148Q seemed to promote IL-18 secretion spontaneously. This was also found in FMF patients with the p.E148Q-M694I allele, whose cells have increased IL-18 secretion between flares, compared with FMF patients who were p.M694I heterozygous [11, 48]. IL-18 can potentiate IFN-γ production, which would be beneficial against intracellular pathogens [49]; however, this remains extremely speculative at this stage.

There are several limitations to this study. Overexpression models such as we employed here do have caveats and could potentially show an artificial effect. However p.E148Q alone or with other FMF mutations showed a highly reproducible change in the ASC speck data. Additionally, known pathogenic mutations in pyrin are robustly recapitulated in this HEK293T cell model, including constitutive activation for the p.S242R mutation and the TcdB triggering effect of the mutations found in the B30.2 domain. We also independently confirmed our ASC speck data by cytokine expression analysis from THP-1 monocytes.

In stimulated HEK293T cells, the contribution of p.E148Q is not seen with p.M694V and only shows a trend for p.M694I (Fig. 4b). In the case of p.M694I, the effect of p.E148Q was shown only in the co-transfection experiment with WT pyrin (Fig. 5). This could indicate a saturation effect in our assay for the potent B30.2 pathogenic variants. Indeed, the effect of p.E148Q was only clearly seen with the moderate mutations p.V726A and p.R761H (Fig. 4b), the second of which was statistically significant, or if the mutated allele was co-expressed with the WT allele (Fig. 5). We suspect that p.E148Q would have a similar effect on the more potent mutations than with the more moderate ones; however, we could not demonstrate this due to the limitations of our assay.

A further limitation of our experimental conditions is that the co-expression experiment used expression vectors tagged with the same fluorophore. However, we have multiple biological repeats, so that reduces the technical variability associated with plasmid concentrations, volumes, and transfection efficiency. In the future, generating additional pyrin plasmids with different flurophores will make more sophisticated analysis feasible.

Finally, it should be stated that diagnosis of FMF is often made by singleton sequencing or commercial panels. This limits the information available to the clinician regarding the presence of a possible pathogenic variant in the *MEFV* gene, with no indication about the position of the variant *in cis* or *in trans*.

In summary, we have shown in this study that p.E148Q has a functional effect favouring constitutive assembly of the pyrin inflammasome. Due to its prevalence in several populations, it could play an important role as a risk factor for inflammatory diseases and for the expressivity of FMF if combined with other pathogenic mutations. For some families, the presence of p.E148Q *in cis* of other FMF pathogenic mutations could increase the risk of dominantly inherited FMF, or otherwise promote disease along with other genetic, epigenetic or environmental factors.

## Supplementary Material

kead376_Supplementary_Data

## Data Availability

All other data is available in the main text, supplementary materials or upon request from the authors.
