# Supplementary material for: Pyrin variant E148Q potentiates inflammasome activation and the effect of pathogenic mutations in cis
Source: Rheumatology (Oxford). 2023 Jul 22;63(3):882–90. doi: 10.1093/rheumatology/kead376 (PMC10907813; doi:10.1093/rheumatology/kead376)
Supplement: kead376_Supplementary_Data [file kead376_supplementary_data.docx]

**Supplementary Material**

**Supplementary Data S1. Clinical Information**

*Clinical information for Family 1*

Family 1 is Lebanese Australian and have three children, two of whom demonstrated an FMF phenotype.  Patient 1 (II:3) is a girl aged 14 who had recurrent episodes of fever with raised inflammatory markers from early in life.  Episodes recurred as frequently as monthly but varied through childhood and with therapy. She was started on colchicine 0.5mg twice daily at age 6 and has weaned this dose to one tablet once daily more recently. Inflammatory markers during episodes were elevated (e.g. ESR = 97mm/hr, CRP = 113mg/L) and when undertreated she also manifested raised interval inflammatory markers when she was well (Serum Amyloid A (SAA) = 236 mg/L (N <4), CRP = 74mg/L, however when her dose of colchicine was increased her interval markers normalised.  Patient 2 (II:2) the brother of patient 1, is aged 11 years. He also had episodes of inflammation associated on occasion with pleuritic chest pain.  His inflammatory markers during episodes have been elevated (e.g. ESR = 34mm/Hr, CRP = 126mg/L, Serum Amyloid A = 816mg/L.  Interval inflammatory markers were normal throughout childhood.  Patient 3 (I:1) the mother of patients 1 and 2 had multiple episodes of fever, occurring up to monthly over many years and associated with abdominal pain on occasions.  Notably, both mother and children struggled to grow quickly during childhood, and all three fall at the lower end of the normal range for stature, with the sister being 149.10cm (2.7^th^ centile) for height and 40.10Kg (5.4^th^ centile) for weight, while her brother is still growing and is 133.60cm (1.7^th^ centile) for height and 26.7Kg (0.6^th^ centile) for weight.  The father (I:2) and 18-year-old brother (II:1) who do not share the phenotype and are both above the fiftieth centile for height. Sanger sequencing of *MEFV* identified that mother and children carry the p.M694I and p.E148Q variants, which are not carried by the father or the older brother, confirming that the variants are *in cis* and that this is a complex allele.

*Clinical information for Family 2*

Family 2 is Egyptian Australian and displays a dominant history of FMF running back through the family.  Patient 1 (III:2) is a 9-year-old boy with two younger sisters (2 years of age and 3 months of age) who presented with episodes of fever and abdominal pain resembling peritonitis, occurring every 3 months, lasting at least a week, between the ages of four and seven.  He is currently on colchicine 0.5mg/twice daily, with occasional breakthrough, and potentially requiring a biologic drug because of intolerance to higher doses of colchicine.  During episodes he had markedly elevated inflammatory markers, (e.g. CRP = 78mg/L and SAA = 1240mg/L).  Interval inflammatory markers settle back down to normal.  Of note his growth has been normal with height on the 50^th^ centile and weight on the 60^th^ centile.  Patient 2 (II:2) the 36-year-old father of P1 developed his first symptoms of clinical FMF in his mid-20s, progressing from short periods of abdominal pain and fevers every 3 months to longer periods of up to a week occurring monthly.  He was started on colchicine and required a dose of 0.5mg twice daily to bring adequate control.  The 64-year-old father of P2 who lives in Egypt has never been diagnosed with FMF, but has a history of abdominal pain and fevers over many years. Sanger sequencing of *MEFV* identified that both proband and father carry the p.M694I and p.E148Q variants. The mother does not carry those variants, confirming that those two variant are in cis.

a.

b.

**Supplementary Figure S1** *Gating strategy for the analysis of ASC speck formation*

(**a**) For the ASC speck assay in HEK293T cells, single lived cells were gated then further separated based on expression of GFP (ASC) and mCherry (pyrin). The cells presenting a speck were then determined based on comparison of GFP width and GFP area. (**b**) To allow comparison, mCherry (pyrin-WT) expressing cells were gated such that 10% of cells contained a speck. The same mCherry (pyrin-WT) gating was then used to evaluate the effect of other mCherry (mutated pyrin) constructs, for example this increases to 52% cells containing specks for p.S242R pyrin.

**Supplementary Figure S2** *Immunoprecipitation of pyrin to evaluate 14-3-3 binding*

GST pull-down assay in HEK293T cells was performed after transfection of WT pyrin or mutated constructs bound to GST. Cells were lysed after transfection and co-immunoprecipitation executed using glutathione Sepharose 4B beads. Blotting for pyrin, pan-14-3-3 and actin after electrophoresis of the whole cell lysate (WCL) on the right and after the co-immunoprecipitation (IP) on the left. p.S242R pyrin did not show 14-3-3 binding after co-immunoprecipitation as expected. No differences were seen in 14-3-3 binding between the p.E148Q and WT pyrin constructs. This result suggested that p.E148Q promotes inflammasome formation spontaneously by another mechanism than the effect of the p.S242R mutation. The graph shows a representative result of 3 independent experiments.
